# The Effect of Aggregate Shape on the Properties of Concretes with Silica Fume

**DOI:** 10.3390/ma13122780

**Published:** 2020-06-19

**Authors:** Jarosław Strzałkowski, Halina Garbalińska

**Affiliations:** Department of Building Physics and Building Materials, Faculty of Civil Engineering and Architecture, West Pomeranian University of Technology Szczecin, al. Piastów 50, 70-311 Szczecin, Poland; Halina.Garbalinska@zut.edu.pl

**Keywords:** aggregate shape, silica fume, thermal properties, compressive strength, basalt aggregate, gravel natural aggregate, mercury porosimetry, optical porosimetry

## Abstract

The paper examines the impact of aggregate shape on the compressive strength and thermal properties of concretes with silica fume based on two different aggregates: natural round gravel aggregate and crushed basalt aggregate. Compressive strength and thermal properties of individual concretes were determined during the first year of specimens curing. Additionally, porosity tests were conducted using mercury intrusion porosimetry and optical porosimetry. Mercury porosimetry tests showed that the use of silica fume led to a decrease in the content of pores of size smaller than 0.15 µm compared to the reference concretes without the addition of silica fume. However, tests carried out on crushed basalt-based concrete showed the presence of numerous additional pores with diameters ranging from 0.05 to 300 μm. In case of natural round gravel aggregate-based concrete, the addition of silica fume brought about an increase in its compressive strength. In turn, basalt-based concrete exhibited notably lower compressive strength values due to significantly higher porosity within the range of more than 70 μm. In basalt concrete, the obtained λ values are much lower than in concretes with normal gravel aggregate. In addition, the specific porosity structure had its impact on the process of drying of specimens of each group which occurred at a significantly faster rate in the basalt-based concrete. In conclusion, it can be stated that the use of crushed basalt aggregate causes a significant aeration of concrete, even despite the use of silica fume. As a result, the concrete based on crushed aggregate is characterized by a definitely lower compressive strength, but also better thermal insulation properties compared to analogous concrete made on natural round gravel aggregate.

## 1. Introduction

The properties of concrete depend on factors such as the type of aggregate used, water to cement ratio, the amount of cementitious materials, or the amount of highly reactive pozzolans such as silica fume. The basic factor affecting the properties of concrete is the use of different types of coarse aggregate [1,2]. Parameters such as aggregate density, its porosity, water absorption, or grain shape and size affect the final parameters of the concrete itself.

### 1.1. Aggregate Shape

Ueno, A. investigated [3] the influence of the particle shape of coarse aggregates on two optimal fine to total aggregate ratio values based on the virtual voids-ratio diagram as well as obtained in an actual concrete compaction experiment. The compaction of the concrete improved as the average coarse aggregate particle shape became more equidimensional.

Ley T. presented [4] the effect of aggregate gradation, volume, and aggregate shape on the concrete properties such as the workability, required paste content, and mechanical properties of a concrete mixture. The distribution of a gradation curve called the Tarantula Curve, which can be helpful to assess the proper aggregate proportions, was presented. The methods of optimization of concrete mix proportions such as coarseness factor, mortar factor, and aggregate particle distribution have been discussed in [5], Larrard F. and Sedran T. investigated [6] different packing models used for the ultra-high-performance concretes.

Larrard T. et al. [7] focused on aggregate shapes on drying and carbonation phenomena [7] by means of 3D finite element simulations. For the drying and carbonation phenomena considered in this study, the influence of the coarse aggregate shapes appears negligible (low for flat shapes) compared to their volume fractions. If more precise values are needed, then the aggregate shape should be considered whenever its aspect ratio is more than about 2, as it may affect noticeably the results.

Jie W. et al. [8] examined an experimental and numerical model describing the effects of the aggregate shapes and exposure duration of chloride diffusion into cement-based materials. The aggregate shape and the exposure duration are of high significance. The model with crushed granite presented a good resistance against chloride ingress, while the model with rounded gravels showed some sensitivity to the chloride penetration.

In Zhou Y. et al. research [9] the results show that the effect of large size aggregate on the crack and strength is more obvious, and the difference in elongation ratio and roundness distribution of aggregate cause the fluctuation of concrete strength. Compared with small-sized aggregate, large-size aggregates have a stronger guiding effect on crack path, which is influenced by aggregate geometry, they increase the uncertainty of material strength in the simulation result. Piotrowska E. et al. [10] focused on identifying concrete behavior under high triaxial loading. The analysis indicated that coarse aggregate shape exerts no influence on the concrete response at high confinement. Irregularly-shaped coarse aggregates slightly increased the overall strength of concrete.

Xiong X. et al. compared the properties of aggregates with sharp and rounded edges [11]. By using shape-modified coarse aggregates with rounded edges, the compressive strengths and bulk densities of the specimens were increased, while the apparent porosity was decreased in comparison to aggregate with sharp edges.

In Sasanipour H. et al. research [12], the durability performance of self-compacting concretes containing coarse recycled concrete aggregates as a partial or total replacement of natural aggregates, and silica fume as a partial replacement of cement, was investigated. Replacing coarse recycled concrete aggregates decreased durability performance, but using silica fume in mixes significantly enhanced the electrical resistivity and chloride ion penetration resistance of self-compacting concrete.

### 1.2. Highly Reactive Pozzolans

Silica fume is one of the most commonly used concrete supplementary cementitious material. Changing concrete microstructure, it affects the basic properties of concrete, impacting both the concrete’s physical parameters (e.g., density, absorptivity) and mechanical characteristics (e.g., compressive strength). It is often used as an reactive pozzolan, in high performance concretes [13,14,15] and in lightweight concretes [16,17,18].

Bhattacharya M. investigated [19] the effect of silica fume on the process of hydration and the properties of cement mortars. The addition of 4% and 8% of silica fume caused an increase in compressive strength in relation to the control sample. In 12% silica fume addition specimen, compressive strength was clearly reduced. In all silica fume variants, the porosity of the mortars was slightly increased.

In the paper [17], the higher the content of silica fume, the higher the volume density of the tested foam concrete. Concretes with high content of silica fume were also characterized by higher compressive strength. Similar results were obtained in [20], which examined the effect of silica fume on concretes with the addition of non-desalinated sea sand.

Wang X. et al. indicated [21] that in the case of foam concrete, the addition of microsilica enables obtaining a more stable mixture with better properties than foam concrete without the microsilica.

Zarnaghi V.N. et al. discussed [22] the effect of silica fume on the properties of light self-compacting concretes of various water to cement ratios. The silica fume content ranged from 0 to 12.5% of the cement mass. The use of silica fume to approx. 5% causes an increase in strength, while its higher amount reduced compressive strength of the specimens. Mercury porosimetry tests showed that only a small amount of silica fume (at 2.5% content) led to a decrease in porosity. The other composites were characterized by higher porosity rates. Similar results were presented in [23]. The authors claim that the silica fume can reduce water absorption and porosity.

Concretes with rubber waste [24], or waste fibers from coconuts [25] were evaluated. In both studies, the use of microsilica enabled to partially mitigate the decrease in mechanical properties of concrete with waste materials. Abd Elhakam A. et al. discussed [26] the improvement of properties of concrete with recycled aggregates through the use of microsilica. Ten percent of silica fume (by weight) increased the compressive and flexural strength of concrete with 75% recycled aggregate content. Similar conclusions were found by others [27,28,29]. The properties of concrete were also influenced by the water to cement ratio. The papers [30,31,32,33] show its effect on the sorption properties of concretes and mortars.

The addition of marble dust [34] alone has a negative effect on the compressive strength of the concrete. However, the use of a mixture of silica fume and marble dust in the total amount of 30% by weight of cement enables to maintain comparable properties to reference concrete without additives.

Research is also being carried out to develop computational models for the evaluation of the strength characteristics of concrete with added silica fume depending on the pozzolan content. A model [35] based on neural networks, allowed an effective determination of the designed parameters of the tested concrete before its formation.

The report [36] attempts to diagnose the scale of variability of the main thermal, moisture, and strength characteristics of silicate products of different humidity rates. The studies conducted show that it is not the total number of pores that is of importance, but their specific geometry. The more small-diameter pores in the material, the greater its durability. It was proved that large capillary pores and air voids have the most adverse effect on material strength properties.

In the study [37], the density of silica fume was increased by producing silica fume granules mixed with a solid super plasticizer. Results indicated an increase in strength and surface electrical resistivity, and a decrease in permeability for both slurry silica fume and granule, compared to the control sample.

The papers [38,39,40] presented the results of studies on concretes with the addition of nanosilica in which the natural quartz aggregate was replaced by glass cullet. Nanosilica was added in the amount of 1% and 3% of cement weight. In both cases, an increase in compressive strength was observed. However, nanosilica caused a deterioration in workability and a reduction of fresh mixture slump. Higher shrinkage was also observed after drying. Additional information about the properties of concrete with silica fume and waste glass has been discussed in [41]. Other supplementary cementitious materials, such as magnetite dust, whose effect on the properties of heavy concretes is shown in [42,43], were also tested.

Li et al. investigated the influence of nanosilica [44]. Authors studied concretes with the addition of both ordinary microsilica and nanosilica. The addition of nanosilica increased the water demand for mixtures and the necessary amount of superplasticizers in order to obtain the same consistency as in concrete without silica additives. The concretes with the same compressive strength as normal nanosilica concrete did not require additional superplasticizers. The most beneficial in terms of strength was the concrete mix containing both ordinary microsilica and nanosilica. Abd Elrahman, M. et al. utilized nanosilica in lightweight concrete [18]. An improvement in strength and a reduction of transport properties, in specimens with an increased nanosilica content, were observed. The positive effects of nanosilica were more pronounced when a higher amount was incorporated into the mixtures (>1 wt.%). Nanosilica contributed to compaction of the lightweight aerated concrete matrix and a modification of the air-void system, by increasing the amount of solid content and refining the fine pore structure, which translated to a noticeable improvement in mechanical and transport properties.

A variety of results presented in this introduction encouraged us to do own research on the influence of coarse-grained aggregate shape on the properties of concretes with the addition of the silica fume. By replacing the ordinary round gravel aggregate with heavy crushed basalt aggregate, we expect to obtain a composite with increased density, higher compressive strength, and better accumulation properties than in normal concrete based of gravel.

Therefore, in this paper, we decided to examine and compare the properties of concrete based on two kinds of aggregate types: natural round gravel aggregate and heavy crushed basalt aggregate. The list of essential objectives of our study is presented below:

- how does the type of coarse aggregate affect pore structure of the whole composite;
- whether the use of aggregate with higher resistance to crushing will increase compressive strength of the concrete;
- what is the impact of the aggregate used on thermal properties of the concrete;
- what is the influence of aggregate of the heat accumulation properties and dynamic heat flow;
- whether the use of high density basalt aggregate will hinder the process of free drying of the material;
- whether the use of silica dust additive will allow additional sealing and compaction of the composite cement matrix.

## 2. Materials and Methods 

The studies were carried out on concretes made with conventional coarse round gravel aggregate (Ref) and crushed basalt aggregate (Bas). All formulas were tested on aggregates of 4–8 mm fraction. Two main concretes were prepared with silica fume in the amount of 8% of cement weight and superplasticizer (0.75% of cement weight) to maintain the desired consistency of the mixtures. The first formula was based on round gravel aggregate (Ref-8) and the second one on crushed basalt aggregate (Bas-8). The additional formula was also produced based on natural round gravel aggregate without any additives or admixtures (Ref-0), as a reference material, in order to determine the effect of silica fume on specific properties of concrete.

**Figure 1:**
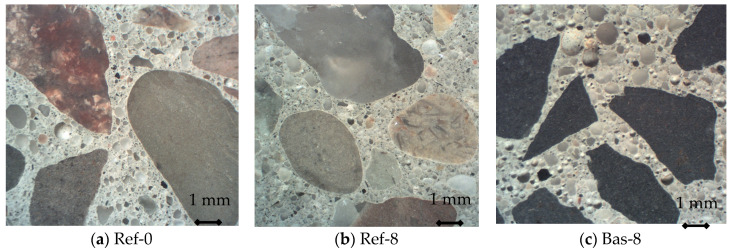
Cross-sections of specimens.

The cross-sections of the specimens are shown in Figure 1. There is a visible difference in the shape of coarse aggregate. Natural gravel aggregate has a rounded contour and compact shape, unlike basalt aggregate with sharp and jagged edges. The basic properties of used aggregates are shown in Table 1.

Composition of individual mixtures is shown in Table 2. All variants used the same cement CEM I-42.5 R. The ratio of water to cement was constant and was 0.55. In case of all mixtures, a constant proportion (1.2) of the amount of 0–2 mm sand to cement was kept.

For all concretes, a total of 26 specimens of size 10 cm × 10 cm × 10 cm were made which were used for the strength and porosimetry tests [45]. Specimens were formed using plastic bisected moulds. Each specimen in the mould at the time of filling was compacted twice, using a vibrating table to ensure adequate filling and concentration of the mix. The specimens were stored for the first 28 days in a high humidity climate chamber (temperature 20 ± 2 °C and humidity > 90%) [46]. Then, the specimens were subjected to free drying in dry laboratory air conditions.

Compressive strength was tested after 7, 14, and 28 days and 3, 6, and 12 months of curing. Four specimens 10 cm × 10 cm × 10 cm were tested at each time for each concrete. For every type of concrete and given test date, the mean value of compressive strength *f_cm_*, standard deviation, coefficient of variation, and measurement uncertainty were calculated. The uniaxial compression strength test module was used. The stresses were uniformly increased according to standard EN 12390-3 [47].

Porosity was analyzed using mercury intrusion porosimetry [48]. After three months of curing, cross-sections were cut from the cubic specimens, which were then used to prepare 0.7 cm × 0.7 cm × 2.0 cm specimens. Glass cells of 2 cm^3^ volume were used. The specimens had approximately 1 cm^3^ volume, which is consistent with the standard ISO 15901-1 [48]. For each material, two specimens were analyzed. Before testing, the prepared specimens were dried in a laboratory dryer until constant weight was obtained. The surface tension of mercury was assumed to be 0.48 N/m and the contact angle was set at 140 degrees for the intrusion. Firstly, specimens were exposed to low pressure (up to 0.34 MPa), then mercury-filled cells with specimens were weighed. Secondly, the specimens were placed in a pressure chamber and subjected to high pressure (up to approx. 413 MPa). On the basis of obtained results of the injected mercury volume, the integral porosity graphs, total porosity (in the research scope of the MIP method) and total specific surface area of specimens were determined.

**Figure 2:**
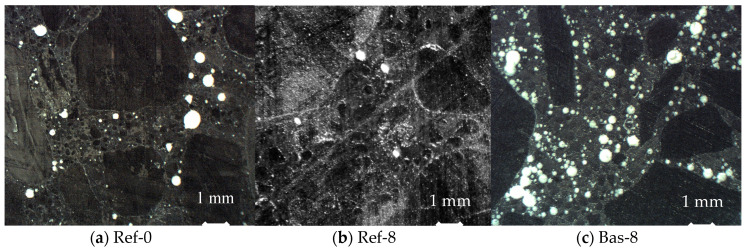
Cross-sections of specimens prepared for optical porosimetry tests.

In addition, optical porosimetry tests were conducted using the automatic system for analyzing the air content in hardened concrete. From the cured cubic specimens, 1 cm thick central cross-sections were cut. The specimens were carefully polished with abrasive powders. Then, the surface of the specimens was painted black and the pores on the surface of the specimens were filled with white zinc paste. Two specimens were tested for each concrete. The analyzed surfaces had an area of 8 cm × 8 cm, while the traverse from which the readings were carried out was 1600 mm long. Each specimen was tested twice. The second reading was performed with the specimen rotated by 90 degrees in the device. Figure 2 shows the specimens’ cross-sections prepared for the tests.

Tests of the changes of thermal parameters were performed using the nonstationary method. This technique is based on the analysis of heat flux readings at its non-stationary flow. The device uses surface probes or needle probes with a certain range of thermal conductivity. For the experiment, the surface probe of range from 0.3 to 2.0 W/mK was used. The probe heats the specimen with a specific heat flux and measures the thermal response in time. Thermal conductivity *λ* and volumetric specific heat *c_v_* were recorded. The tests were performed during the first 12 months of specimen curing, i.e. after 7, 14, and 28 days, and after 2, 3, 6, 8, 10, and 12 months. The testing was carried out on six specimens made from each type of concrete. The specimens were 4 cm × 14 cm × 16 cm in size, and the analyzed area was the bottom base of the specimen. A measuring location was marked on each of the six specimens to get the parameters from exactly the same place on the specimen each time. The mean values and standard deviations of the results were determined based on the measurements. After a year of curing, the tests were additionally carried out on the specimens dried at the temperature of 70 °C to a constant mass.

**Figure 3:**
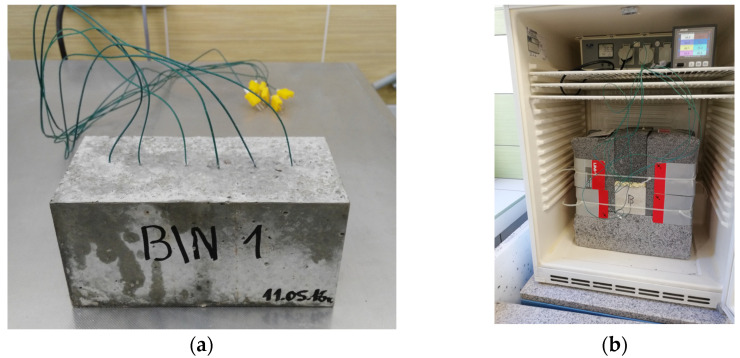
Example specimen (**a**) and the test stand (**b**).

Additional tests were performed to record the temperature of specimens subjected to an abrupt change in ambient temperature. For this purpose, rectangular specimens with a cross-section of 10 cm × 10 cm and a length of 20 cm were prepared. A set of K-type thermocouples (measurement accuracy +/− 0.75%) was embedded in the longitudinal axis of the specimen during its formation. The specimens prepared in this way were demolded after 24 h and stored on a grate in a water bath with increased humidity for 28 days. The specimens were then dried at 70 °C until constant weight was achieved. Then, the specimens were insulated on the side surface with graphite polystyrene insulation of 15 cm thick and λ = 0.04 W/mK. This made it possible to obtain heat flow close to unidirectional. The example specimen and the test stand are shown in Figure 3.

The specimens prepared in this way were placed in temperature-controlled thermostatic chambers. In the first step, the chamber temperature was stabilized at 10 °C, maintaining it for about 30 h. Then, the chamber temperature was raised abruptly to 40 °C. The eight-channel data loggers were used to collect temperature data. Data was read at a frequency of one reading per minute. Based on the temperature recordings, the heat flux densities on the uninsulated surface and at a depth of 3.4 cm from the surface were calculated.

Moisture content tests were conducted. The plate specimens 4 cm × 14 cm × 16 cm (6 specimens for each material) were weighed during the first 12 months of specimen curing, i.e. after 7, 14, and 28 days, and after 2, 3, 6, 8, 10, and 12 months. After one year, the specimens were dried to constant mass. Based on the differences in mass readings, the moisture content and relative mass reduction were determined. The mean values and standard deviations of the results were calculated based on the measurements.

## 3. Results and Discussion

**Figure 4:**
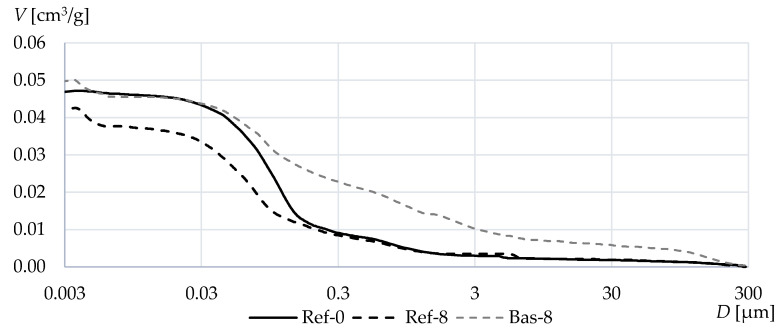
Cumulative normalized volume of the tested concretes measured by means of mercury porosimetry.

Figure 4 shows the integral distribution curves of the pores in different concretes. The use of silica fume led to the decrease in the content of pores of less than 0.15 µm compared to the gravel-based Ref-0 concrete without the addition of silica fume. On the other hand, numerous additional pores in the range from 0.05 to 300 µm were found in the basalt-based concrete. This is due to the use of crushed aggregate which, when mixing the fresh concrete, caused aeration of the cement paste.

In the case of variants with microsilica, additional pores with a diameter below 5 nm also appeared, which did not occur in concrete Ref-0. Generally, in case of concretes with silica fume, the pore graph is slightly more evenly distributed in comparison to Ref-0.

**Figure 5:**
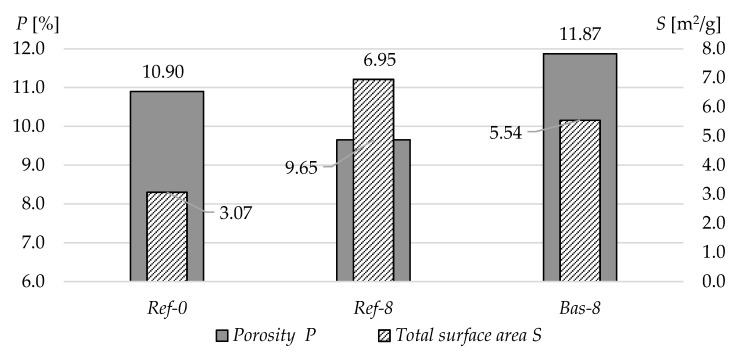
Porosity and total surface area of the tested concretes measured by means of mercury porosimetry.

Figure 5 shows the values of porosity *P* and the total specific surface area *S* obtained from the mercury porosimetry tests. The addition of silica fume in both cases greatly increased the specific surface area. In contrast, the differences in total porosity in terms of mercury porosimetry tests are quite insignificant, pointing to a slight increase in the case of crushed basalt-based concrete and a reduction in conventional gravel aggregate concrete. The obtained results suggest that in the case of basalt concrete, the total porosity only slightly increases, but at the same time, this concrete is characterized by pores with much larger diameters. The use of silica fume did not significantly affect the number of pores with diameters in the range from 0.05 to 300 µm formed during the mixing of fresh concrete. In case of the composites, the silica fume, and superplasticizer, there is also an increase of very fine pore smaller than 5 nm, which do not occur in Ref-0. Probably the increase of these pores increased the total surface area.

**Figure 6:**
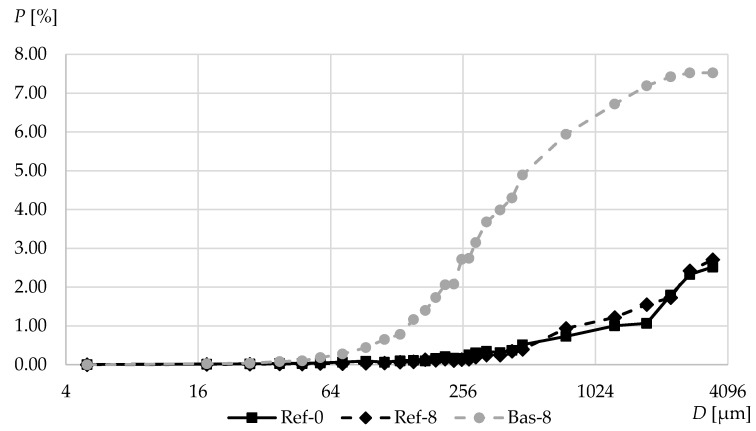
Cumulative air content of the tested concretes measured by means of optic porosimetry.

Figure 6 shows the cumulative porosity graphs obtained using the optical method. In the range of more than 70 μm in the basalt-based concrete, there were numerous pores that were not observed in gravel aggregate concretes. In turn, the differences in both gravel aggregate-based concretes (Ref) are small, and in this respect, silica fume did not lead to a decrease in the pore content. It means that pores which occurred in Bas-8 concrete over 70 µm in size appeared as a result of mixing fresh concrete mix. These pores were created by aeration of the cement matrix caused by the shape of the aggregate itself. In contrast to the gravel round aggregate (Ref), the broken sharp edges of the basalt aggregate caused air entrapment in the cement paste.

**Figure 7:**
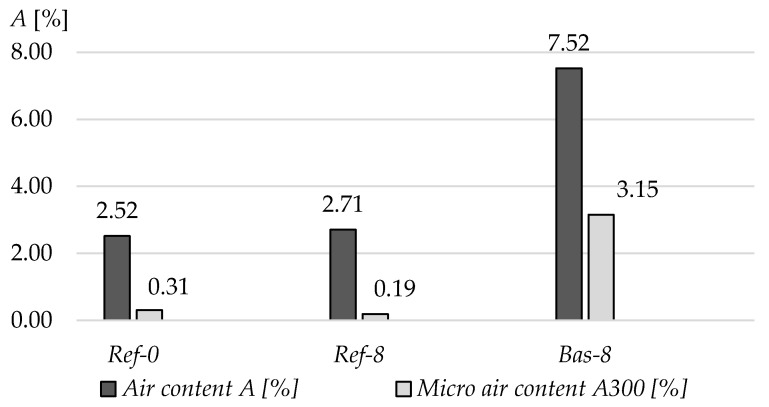
Air content of the tested concretes examined by means of optic porosimetry.

Figure 7 shows the values of total porosity *A* in terms of optical porosimetry and the parameter *A_300_*, which determines the air content in pores of up to 0.3 mm in diameter. A clear difference can be seen in the results for both types of aggregates. Both in the range up to 300 µm and in higher ranges, basalt aggregate concrete was characterized by much higher porosity. The Bas-8 concrete contains numerous additional pores with large diameters in the cement matrix. Most likely this is due to cement paste aeration during the mixing process. This is confirmed by the photos shown in Figure 1 and Figure 2, clearly showing the numerous pores in the crushed basalt-based concrete. What is more, the additional pores have a very round bubble shape—this kind of pore is created in the mixing process.

It seems that the addition of the silica fume and superplasticizer does not influence much the occurrence of large pores—the graphs for Ref-0 and Ref-8 are very similar. Each of these graphs have been created and merged from the results of four specimen optical tests, therefore the measurement uncertainty of the results is small. The high number of large pores which occurred in Bas-8 (in comparison to Ref-8 and Ref-8) is the result of the mixing process of sharp crushed basalt aggregate. If it were otherwise, the differences between Ref-0 and Ref-8 would be much larger.

It should be noted, a significant difference in the results depending on the adopted method of measuring the porosity. In the case of mercury intrusion porosimetry, pores larger than 300 μm are outside the test range. Optical porosimetry, in turn, allows observation of millipores, while pores below 5 μm are invisible for this method. This translates into significant differences in the obtained results. In the case of basalt-based concrete, the optical method has enabled the determination of the extent of occurrence of large millipores that clearly dominate in this concrete.

**Figure 8:**
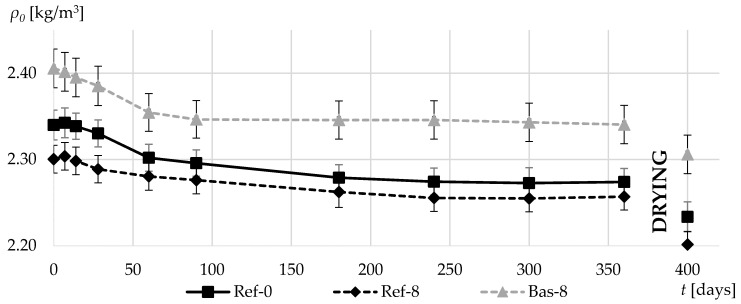
Specific gravities in the first 360 days of composites curing and after additional drying.

Figure 8 shows the change in the specific gravity of concretes during the first year of their curing. For basalt-based concrete, the specific gravity was noticeably higher throughout the whole measurement period, which is due to the significantly higher density of the aggregate itself. The addition of silica fume caused a slight decrease in concrete specific gravity in case of conventional gravel aggregate concrete.

**Figure 9:**
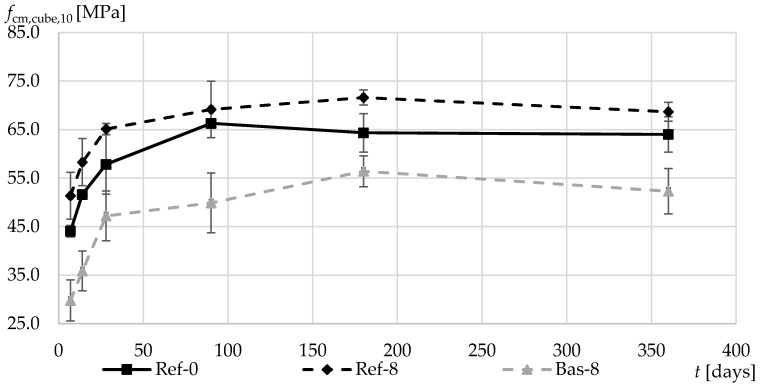
Average compressive strength in the first 360 days of composites curing.

Figure 9 shows the average compressive strength values for cubic specimens determined within the established timeframe of 7, 14, and 28 days and 3, 6, and 12 months after specimen formation. While in the gravel-based concrete, the addition of silica fume led to an increase in the compressive strength throughout the tested period, the basalt-based concrete, due to its definitely higher porosity of the cement matrix in the range of more than 70 μm, was proved to have significantly lower strength values in each of the established measurement periods. It should be noted that basalt aggregate has a much higher mechanical strength (resistance to fragmentation) [1] than natural gravel aggregate. Despite this, the more aerated cement matrix in the Bas-8 concrete resulted in lower compressive strength than in the case of Ref-8 concrete.

**Figure 10:**
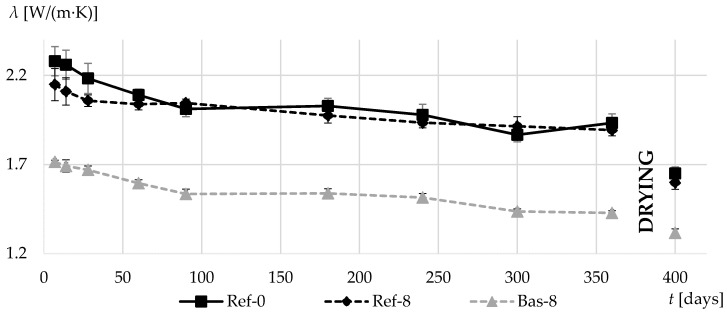
Thermal conductivity coefficients in the first 360 days of composites curing and after additional drying.

**Figure 11:**
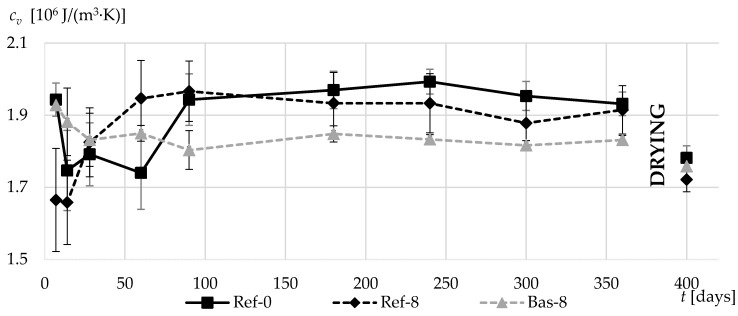
Volumetric specific heat coefficients in the first 360 days of composites curing and after additional drying.

Differences in porosity have also caused changes in the thermal properties of concrete. Figure 10 and Figure 11 present data on the results of thermal conductivity coefficient *λ* and the volumetric specific heat *c_v_*. In Bas-8 concrete, the obtained *λ* values are much lower than in concretes on normal gravel aggregate. In the case of dried specimens, the thermal conductivity drop in comparison to Ref-0 concrete was 20%. The reason for such a decrease in thermal conductivity is the definitely higher porosity of this concrete and in particular cement matrix.

The difference in average results of Ref-0 and Ref-8 concretes is visible only during the first two months of curing. As the specimen moisture content decreases, the differences between Ref-0 and Ref-8 also decreased.

The thermal conductivity of basalt aggregate is higher than gravel. This means that the results of the thermal conductivity of basalt-based concrete Bas-8 should have higher values than the thermal conductivity of the same concrete based on gravel round aggregate Ref-8, but it is not. This means that there are other factors that have a greater impact on the conductivity than the thermal conductivity of the aggregate itself. This factor is the thermal conductivity of the cement matrix itself, which in the case of Bas-8 concrete, is definitely more aerated than Ref-8 cement paste, and as a consequence its thermal conductivity, is definitely lower and affects the resultant conductivity of the entire composite.

During the first three months of concrete curing, the obtained specific heat results were disturbed due to the high moisture content. After this time, the values began to stabilize.

The addition of silica fume in both cases caused a decrease in volumetric specific heat between 90 and 360 days of concrete curing. This is particularly evident in concrete made on the basis of basalt. After drying the specimens to a constant mass, the differences in the specific volumetric heat values are much lower. However, further lower values were recorded for concretes with silica fume.

A slight effect of the density of basalt aggregate (Bas-8) is also visible. In comparison to the Ref-8 after drying, the gravel-based concrete was characterized by approx. 2% lower specific heat, despite the aeration of the matrix in Bas-8 composite.

The thermal dynamics of the material depends both on its thermal conductivity and specific heat. Therefore, it was decided to test the differences in the thermal behavior of individual concrete, by analyzing the heat flux densities.

**Figure 12:**
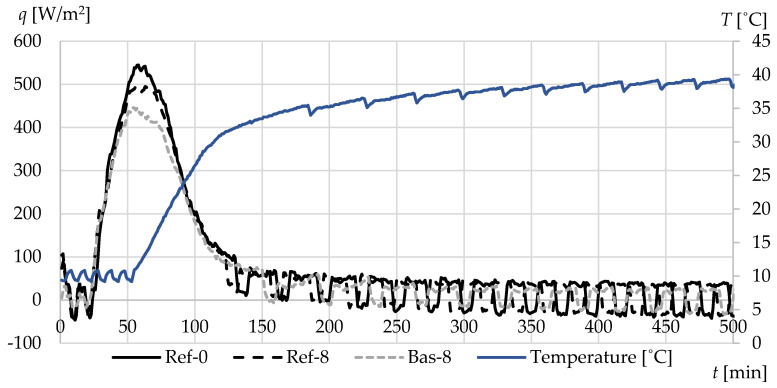
The average heat fluxes on the surface of specimens during heating from 10 to 40 °C.

**Figure 13:**
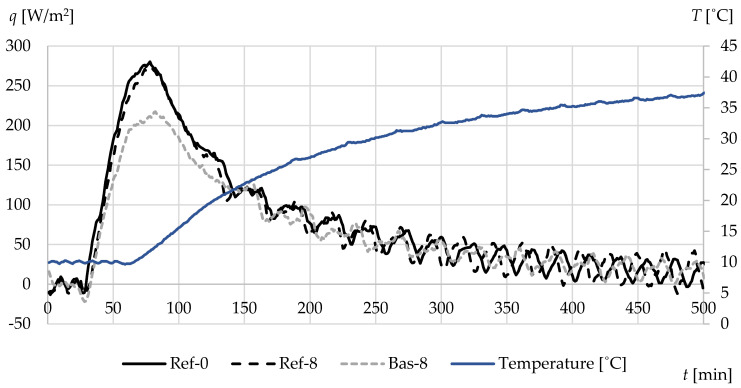
The average heat fluxes at a depth of 3.4 cm during heating from 10 to 40 °C.

Figure 12 and Figure 13 show the values of average heat fluxes densities—on the surface of the tested specimen (Figure 12) and at a depth of 3.4 cm from the uninsulated face of the specimen (Figure 13). The observed increase in heat flux densities occurred as a result of a step change in ambient temperature from 10 to 40 °C. There are visible differences in the maximum values of individual types of concrete, both in relation to the uninsulated lateral surface of the specimens and the densities occurring at a depth of 3.4 cm.

The highest peak was obtained in the case of reference concrete Ref-0, while definitely lower heat flux densities were recorded for basalt-based concrete. This is mainly due to the significantly lower value of both the thermal conductivity and specific heat of this concrete.

**Figure 14:**
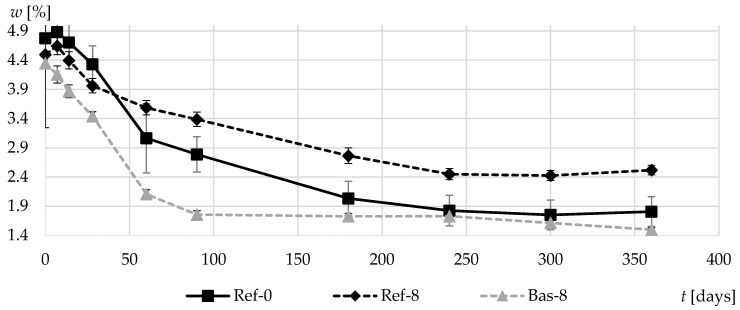
Moisture content of composites in the first 360 days of curing.

Figure 14 shows the change in average moisture content of specimens during the first year of curing. The fastest drying rates were observed in Bas-8 concrete characterized by high content of millipores. In the case of this concrete, it took about three months to stabilize the specimen weight. In turn, for natural gravel aggregate-based concrete, the process took at least 8 months. In the Ref-8 concrete, with the tightest matrix of all the tested specimens, moisture content was the highest after the one-year period. The results obtained differ significantly from those presented in [7]. The indirect effect of aggregate shape on specimen drying proved to be quite significant.

**Figure 15:**
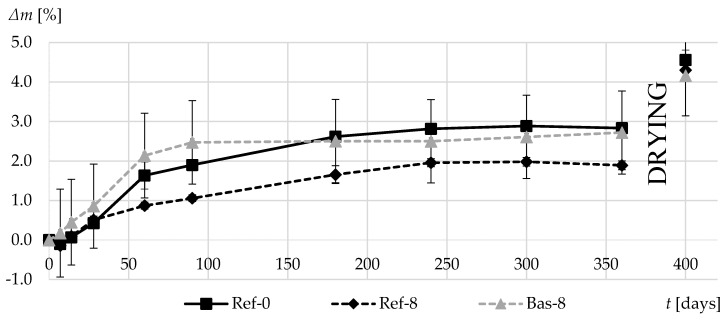
Average relative mass reduction of composites in the first 360 days of curing.

Figure 15 shows the relative weight losses of the tested concretes during the first year of curing. In Ref-8 concrete, with the tightest matrix among those tested, the weight loss was the lowest after a year. Again, the fastest increase in weight loss is characterized by basalt-based concrete with the most porous cement matrix. However, the total relative weight loss values obtained after drying the specimens at 70 °C were similar in all tested materials.

**Figure 16:**
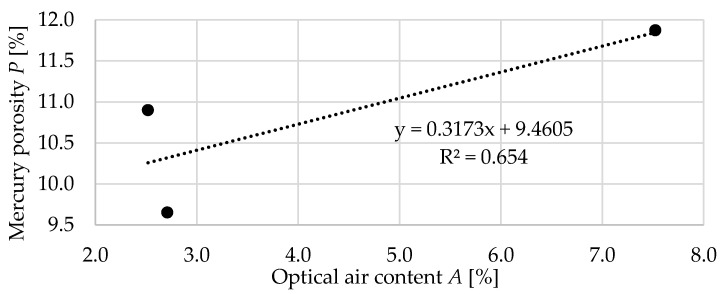
The relation between the optical air content *A* and mercury intrusion total porosity *P*.

Figure 16 shows the relation between the optical air content *A* and mercury intrusion total porosity *P*. As mentioned earlier, the results differ significantly. Despite this, there is a not very strong correlation between the results. The mercury method seems to be effective for pore analysis below 1 um, while in the case of optical analysis, it allows the determination of pore contents larger than 10 um [49,50].

**Figure 17:**
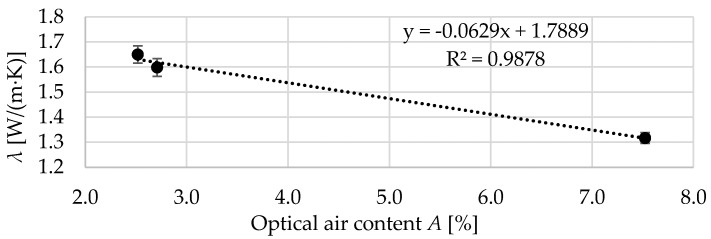
The relation between the optical air content *A* and the thermal conductivity *λ*.

In Figure 17, the relation between the optical air content *A* and the thermal conductivity *λ* is shown. A very strong correlation was observed, for which the coefficient of determination was close to 0.99. No correlation was found for specific heat *c_v_*. This is most likely due to the fact that the used aggregates had different densities, which significantly affected the specific heat as well as aeration of the cement matrix in Bas-8 concrete. The obtained results confirm the conclusions from the work [3]: The compactibility of the concrete improves as the average coarse aggregate particle shape becomes more equidimensional.

**Figure 18:**
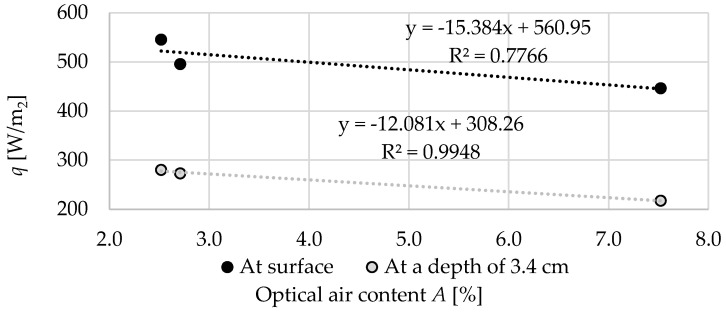
The relation between the optical air content *A* and maximum heat flux density *q*.

Figure 18 shows the relation between the optical air content *A* and maximum values of heat flux density *q*. Quite high correlations were observed between the porosity and the peak of the heat flux density. Especially in the case of a thermocouple located 3.4 cm from the uninsulated surface of the specimen. Surface thermocouple results may be subject to greater inaccuracies due to surface heat exchange effects, which is why the coefficient of determination was lower in this case.

**Figure 19:**
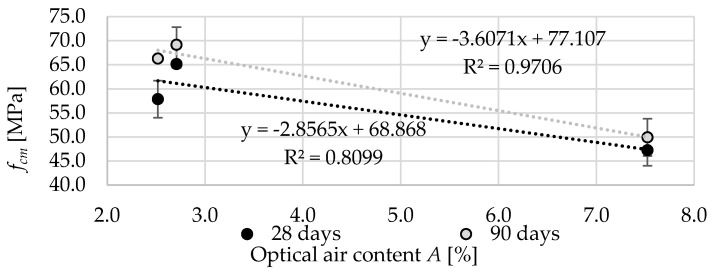
The relation between the optical air content *A* and compressive strength *f_cm_*.

Figure 19 shows the relation between the optical air content *A* and compressive strength *f_cm_* after 28 and 90 days of curing. At both time intervals, the coefficient of determination is quite high, especially in relation to the results after 90 days of curing. Obviously, with the increasing porosity of the concrete, the compressive strength decreased significantly. In contrast to [10], the use of crushed aggregate resulted in a decrease in strength due to weakening of the cement matrix.

## 4. Conclusions

This study examines the impact of aggregate shape on the compressive strength and thermal properties of concretes with silica fume made based on two different aggregates: natural round gravel aggregate and crushed basalt aggregate. The results of the experimental studies can be summarized as follows:

- Additional pores were created by aeration of the cement matrix caused by the shape of the basalt aggregate. In contrast to the gravel round aggregate (Ref), the broken sharp edges of the basalt aggregate caused air entrapment in the cement paste in the Bas-8 concrete.
- The additional porosity of Bas-8 concrete caused a significant reduction in compressive strength compared to reference concretes, despite the fact that basalt aggregate has a much higher resistance to fragmentation than natural gravel aggregate.
- Both the thermal conductivity coefficient and volumetric specific heat have been lowered in concrete based on basalt aggregate in comparison with the data obtained for reference concrete, despite the higher thermal conductivity of basalt aggregate compared to gravel aggregate.
- The process of specimen drying occurred considerably faster in basalt-based concrete despite its higher density exhibited by this composite throughout the entire drying period.

Therefore, it should be concluded that the attempt to improve the mechanical and heat accumulation properties of concrete through the use of a high-quality crushed basalt aggregate and addition of silica fume failed. Despite the fact that, the obtained composite had higher density, the values of compressive strength and specific heat deteriorated.

The total porosity values were increased (especially in range of millipores), which led to a noticeable reduction in the compressive strength of the modified material and also decrease of the values of thermal conductivity and specific heat.

The results point to the need for great caution in predicting the effects on strength and thermal properties associated with the shape of the coarse aggregate and simultaneous use of silica fume, which in some cases may turn out to be not necessarily beneficial.

## Figures and Tables

**Table 1:** Basic properties of natural round gravel aggregate (Ref) and crushed basalt aggregate (Bas).

Aggregate Type	Water Absorption [%]	Specific Gravity [g/cm^3^]	Loose Bulk Density [g/cm^3^]	Compacted Bulk Density [g/cm^3^]
Ref	1.64	2.50	1.54	1.68
Bas	1.19	2.68	1.47	1.71

**Table 2:** Concrete recipes prepared for testing.

Type	Type of Aggregate	Coarse Aggregate	Coarse Aggregate	Sand	Cement	Water	Silica Fume	Super-Plasticizer
		[kg/m^3^]	[dm^3^/m^3^]	[kg/m^3^]	[kg/m^3^]	[kg/m^3^]	[%]	[%]
**Ref-0**	Natural Gravel 4–8 mm	1295.0	488.7	463.0	385.5	212.0	0.0	0.00
**Ref-8**		1269.0	478.8	463.0	385.5	212.0	8.0	0.75
**Bas-8**	Basalt 4–8 mm	1370.0	478.8	463.0	385.5	212.0	8.0	0.75
